# A Model for Structured Information Representation in Neural Networks of the Brain

**DOI:** 10.1523/ENEURO.0533-19.2020

**Published:** 2020-05-29

**Authors:** Michael G. Müller, Christos H. Papadimitriou, Wolfgang Maass, Robert Legenstein

**Affiliations:** 1 Institute of Theoretical Computer Science, Graz University of Technology, Graz 8010, Austria; 2 Computer Science Department, Columbia University, New York, NY 10027

**Keywords:** assemblies, cognition, factorized codes, Hebbian plasticity, spiking neural networks, structural knowledge

## Abstract

Humans can reason at an abstract level and structure information into abstract categories, but the underlying neural processes have remained unknown. Recent experimental data provide the hint that this is likely to involve specific subareas of the brain from which structural information can be decoded. Based on this data, we introduce the concept of assembly projections, a general principle for attaching structural information to content in generic networks of spiking neurons. According to the assembly projections principle, structure-encoding assemblies emerge and are dynamically attached to content representations through Hebbian plasticity mechanisms. This model provides the basis for explaining a number of experimental data and provides a basis for modeling abstract computational operations of the brain.

## Significance Statement

High-level cognition in the human brain necessitates dynamically changing structured representations of information. There exists experimental evidence that in cortex, sensory content is enriched with structural information using a factorized code. We introduce the concept of assembly projections, a general principle for attaching structural information to content in generic neural networks. Assembly projections provide the basis for explaining a number of experimental findings. In addition, the model is capable of performing elementary cognitive processing operations, thus extending the computational capabilities of neural network models in the direction of cognitive symbolic computations.

## Introduction

The flexibility of human behavior is rooted in the ability to assign abstract structural information to sensory content. It has been argued that such handling of structural knowledge is essential for learning on limited training data, for generalization beyond simple interpolation of observations, and for real-world language understanding (Marcus, 2003, 2018; Behrens et al., 2018; Frankland and Greene, 2019a). However, the underlying neural processes that enable flexible assignment of structural information to content have remained unknown.

It is widely believed that sparse assemblies underlie the representation of content in cortex. “Concept cells” have been found in the medial temporal lobe (Quian Quiroga, 2016), which respond to unique concepts present in the stimulus regardless of the mode of presentation, giving rise to a sparse and highly invariant representation of input content. There exists experimental evidence that in cortex, such content is enriched with structural information using a factorized code, i.e., sensory content and structural information are not represented in an intermingled manner, but rather separately. Structural information may be of a continuous nature, such as the spatial or temporal context (Miller et al., 2013; Buzsáki and Tingley, 2018; Behrens et al., 2018), or non-continuous categorical such as semantic roles of objects in episodes (Frankland and Greene, 2019a). In this article, we ask how the latter type, categorical structural information, can be attached to sensory content. Findings of Frankland and Greene (2015) provide some insights into the underlying cortical mechanisms in the context of human language processing. They investigated the cortical response to simple sentences using functional magnetic resonance imaging (fMRI) and found a subarea of the left mid-superior temporal cortex (lmSTC) from which the identity of the agent in the sentence could be decoded. The patient could be decoded from another, distinct part of lmSTC. This indicates that there exist a number of distinct areas in lmSTC, each of which representing structural information of a specific category related to the current stimulus. Analogous findings for the interpretation of visual scenes suggests that this is a more general processing principle (Wang et al., 2016).

**Figure 1.**
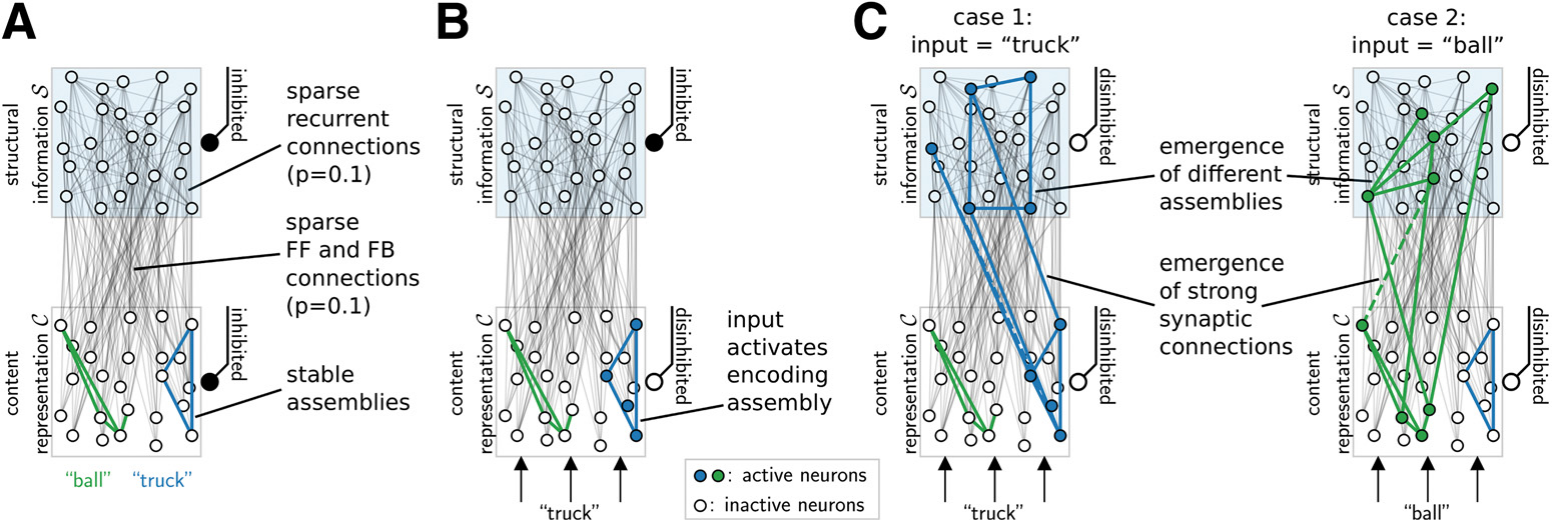
Neural spaces and assembly projections. ***A***, Network structure. Rectangles indicate content space C (light blue, top). Circles denote neurons (open: inactive; filled: active). Disinhibition is shown on the right of each space (filled black circle: inhibited; open circle: disinhibited). Concepts are encoded in the content space through stable assemblies (only two assemblies for two contents “ball” and “truck” are shown, strong recurrent connections are shown with colors based on assembly identity). Thin gray lines between C and S as well as within S show sparse feedforward (FF), feedback (FB), and recurrent connections (randomly initiated, connection probability *p *=* *0.1). Only a subset of 25 neurons is shown per space. ***B***, Presentation of a concept through input neurons (bottom, arrows) activates the encoding assembly in the content space if it is disinhibited (filled circles: active neurons). Initially, the neural space S is inhibited, preventing plasticity from occurring at the potential feedforward/feedback and recurrent connections. ***C***, Disinhibition of the neural space enables the activation of neurons there and results in the formation of an assembly projection. Hebbian plasticity at the potential connections leads to the emergence of an assembly in the neural space (strong recurrent connections in S are shown in color), which has strong synaptic connectivity with the active assembly in C (solid colored lines: feedforward connections, i.e., from C to S; dashed: feedback). This mechanism allows the assignment of values to the variable encoded by the neural space. Assignment of different values (left: “truck,” right: “ball”) give rise to different assembly projections (i.e., different assemblies in S and different connectivity between S and C, color encodes identity of the value assigned to the neural space).

We show that these hints from experimental data suggest a novel concept for the general task of attaching structural categories to content and the flexible use of this structured representation in mental tasks: assembly projections. According to this concept, structure-encoding assemblies emerge in distinct subareas for different structural variables and are attached to content representations through Hebbian plasticity mechanisms in generic spiking neural networks. We base our model on a minimal set of assumptions, in particular, we make no assumptions on specific wiring or symmetric connectivity. We model each structure-encoding subarea (referred to as “neural space” in the following) as one population of neurons with divisive inhibition (Carandini and Heeger, 2011; Wilson et al., 2012). The full model may have several such neural spaces, one for each represented structural category. These neural spaces are sparsely connected to a single “content space” where neural assemblies represent sensory content, akin to concept cell assemblies (Fig. 1*A*). Such a context-invariant representation has been proposed previously, perhaps situated in the posterior middle temporal gyrus or broadly distributed (Frankland and Greene, 2019a). We propose that the control over the attachment of structural information to content is implemented through disinhibition (Pfeffer, 2014; Caroni, 2015; Harris and Shepherd, 2015). When a neural space for structural information is disinhibited while a content assembly is active, a sparse assembly also emerges there. Fast Hebbian plasticity ensures that this assembly is stabilized and synaptically linked to the content assembly. In other words, the neural space for structural information now contains a projection of the content assembly, an assembly projection. We propose that information about this assembly is retained in the neural space through transiently increased neuronal excitabilities (Letzkus et al., 2015).

**Figure 2.**
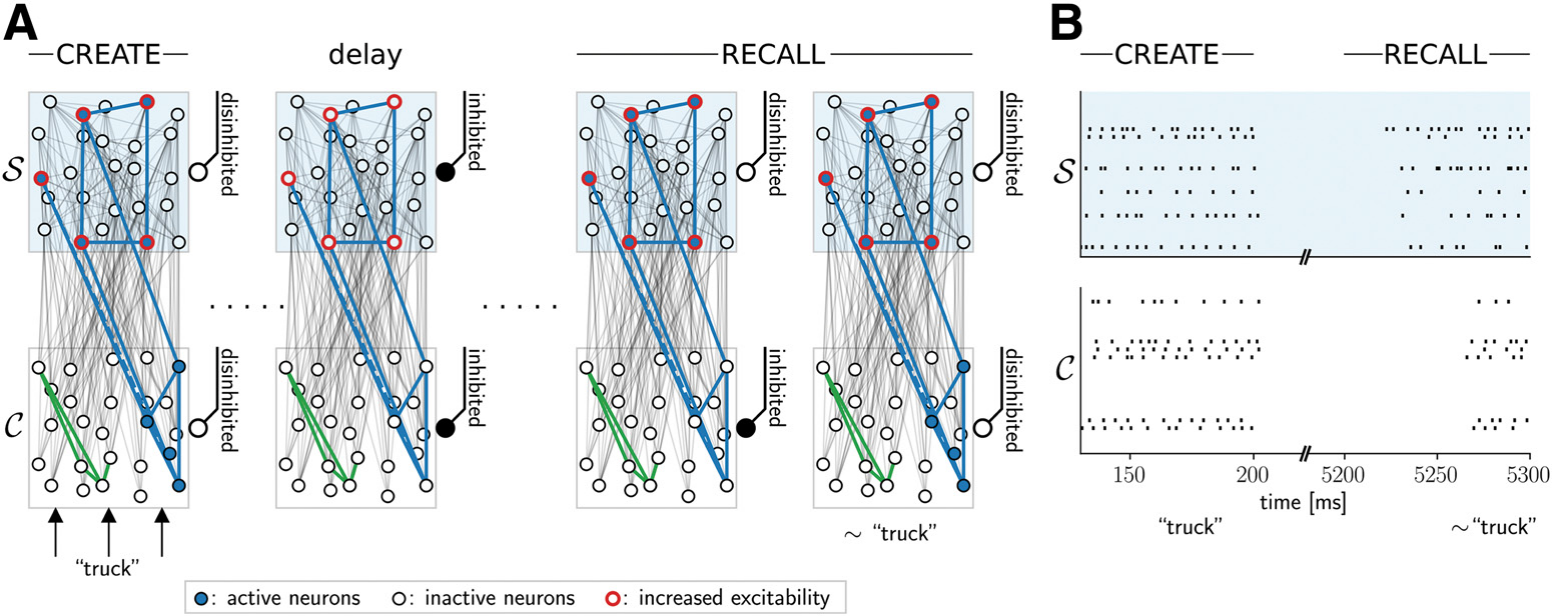
Recall through assembly projections. ***A***, Recall of a previously created assembly projection (schematic drawing). After an assembly projection was formed (CREATE) for the word “truck,” the excitability of assembly neurons in the neural space S is enhanced (indicated by red color). When the neural space is disinhibited, these neurons are activated, and in turn, they activate the “truck” assembly in content space C (RECALL). ***B***, Spike rasters from neural space S (top) and content space C (bottom) in a simulated recall (only 20 randomly selected neurons shown per neural space for clarity). After a CREATE (left, up to 200 ms), and a delay for 5 s, a RECALL is initiated by first disinhibiting the neural space S (at time *t *=* *5200 ms) and then disinhibiting the content space C (50 ms later).

**Figure 3.**
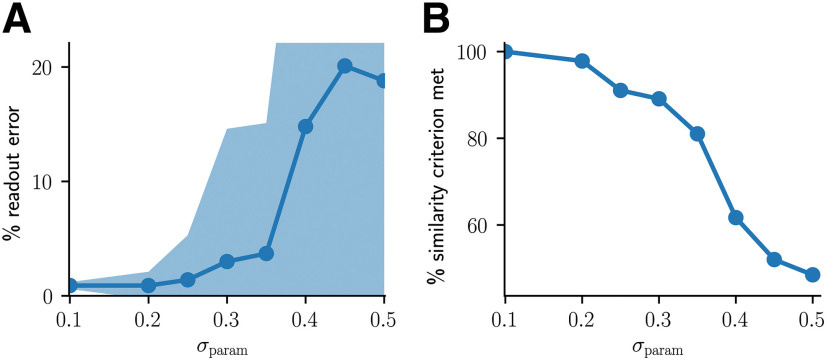
Robustness of RECALL performance to parameter variations. Twenty of the model parameters controlling plasticity (see Table 2, all highlighted parameters except for *α* values controlling the overall shape of STDP curves) were randomly varied by sampling each parameter value *v* from normal distributions with mean *v* and SD $\sigma_{\text{param}}v$. ***A***, Resulting readout classification error on the RECALL operation for increasing *σ*_param_. Shown are mean errors (dots) and SDs (shaded area) for 100 randomly sampled parameter combinations per value of *σ*_param_. Even for substantial parameter changes, the readout error remains small. ***B***, Percentage of trials which met the similarity criterion for increasing *σ*_param_.

**Table 2:** Connection parameters for all plastic connections in the model

Connection	Probability	Synaptic delay	Synaptic weight		Plasticity parameters				
			Initial	Bounds	*α*	$\tau_{+}$	$\tau_{-}$	$A_{-}$	*η*
		ms	pA	pA		ms	ms		
Content space									
X→E	1	1, 10	0, 0.8	0, 0.8	0	25		0.4	0.01
S→E	0.1	1, 10	0.19^*^, 0.39^*^	0, 0.87^*^	0^*^	20^*^		0.47^*^	0.008^*^
E→E	0.1	1	0	0, 0.6	–1	25	40^*^	0.5	0.0025
Neural space									
C→E	0.1	1, 10	0.48^*^, 0.86^*^	0, 1.33^*^	0^*^	21^*^		0.28^*^	0.004^*^
E→E	0.1	1	0.44^*^, 0.87^*^	0, 1.08^*^	–1^*^	37^*^	49^*^	0.52^*^	0.006^*^

The parameters are given for incoming connections to the excitatory neurons (E) within the content space from the input population (X) and from neural spaces (S) as well as for recurrent connections within the excitatory pool in the content space. For neural spaces, the parameters for incoming connections from the content space (C) and for recurrent excitatory connections are given. Synaptic delays and initial weights are drawn from uniform distributions within the given bounds. Highlighted parameters (*) were determined using an optimization procedure (see text).

**Figure 4.**
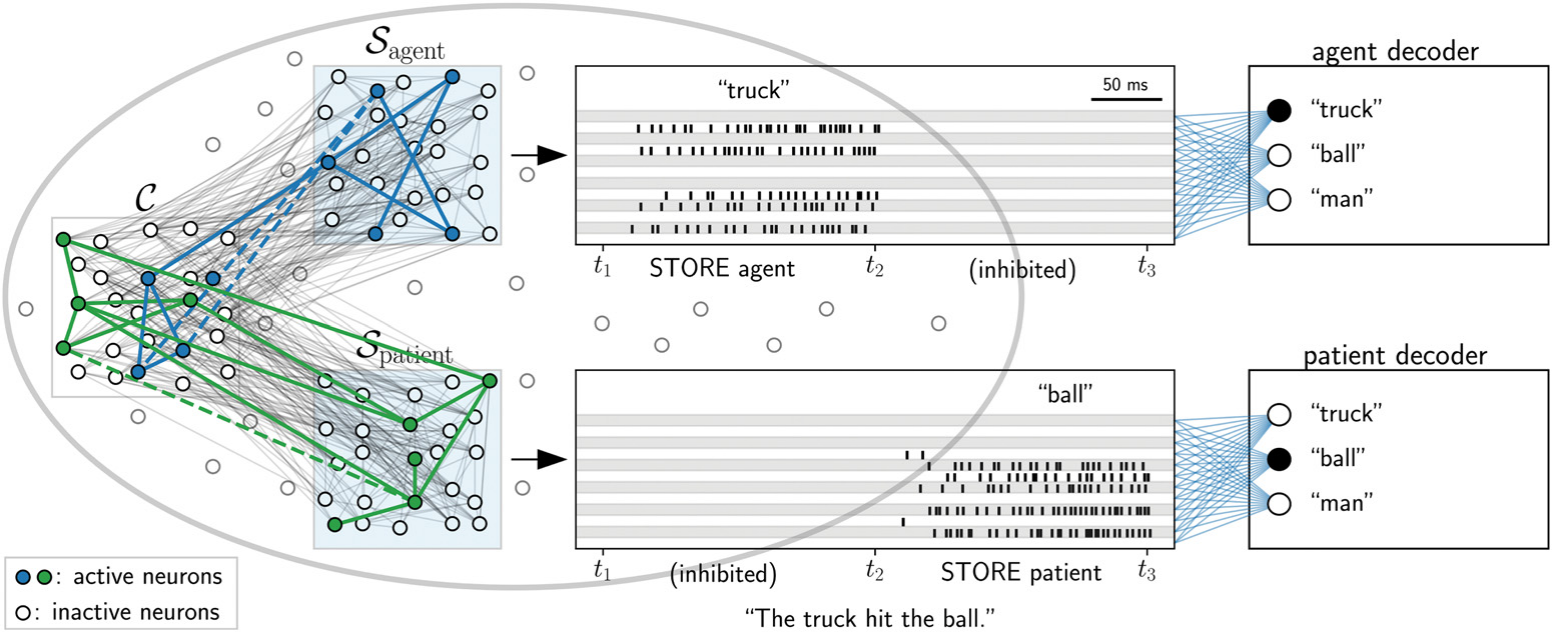
Decoding of agent/patient identity from assembly projection, as performed by Frankland and Greene (2015). Our model of structured information representation in the temporal lobe (gray oval) consists of one content space C (white background, left) and two neural space (light blue background). Neural spaces encode agent ($S_{\text{agent}}$, top) and patient ($S_{\text{patient}}$, bottom) in a sentence (as specific subregions of lmSTC do; see Frankland and Greene, 2015). Presentation of a word leads to the activation of the corresponding assembly in C (only the words for the agent and the patient were processed, verbs and articles were ignored). Words were presented in the order in which they appear in the sentence (for “The truck hit the ball,” the input “truck” was presented while the agent space was disinhibited between *t*_1_ and *t*_2_, likewise disinhibition of the patient space while “ball” was presented between *t*_2_ and *t*_3_). This resulted in the establishment of assembly projections within the neural spaces for agent and patient (insets show spike rasters of a subset of neurons from each neural space). Linear classifiers (“agent decoder” and “patient decoder”) were able to determine the current agent and patient in sentences from the corresponding neural spaces, modeling experimental results (Frankland and Greene, 2015).

**Figure 5.**
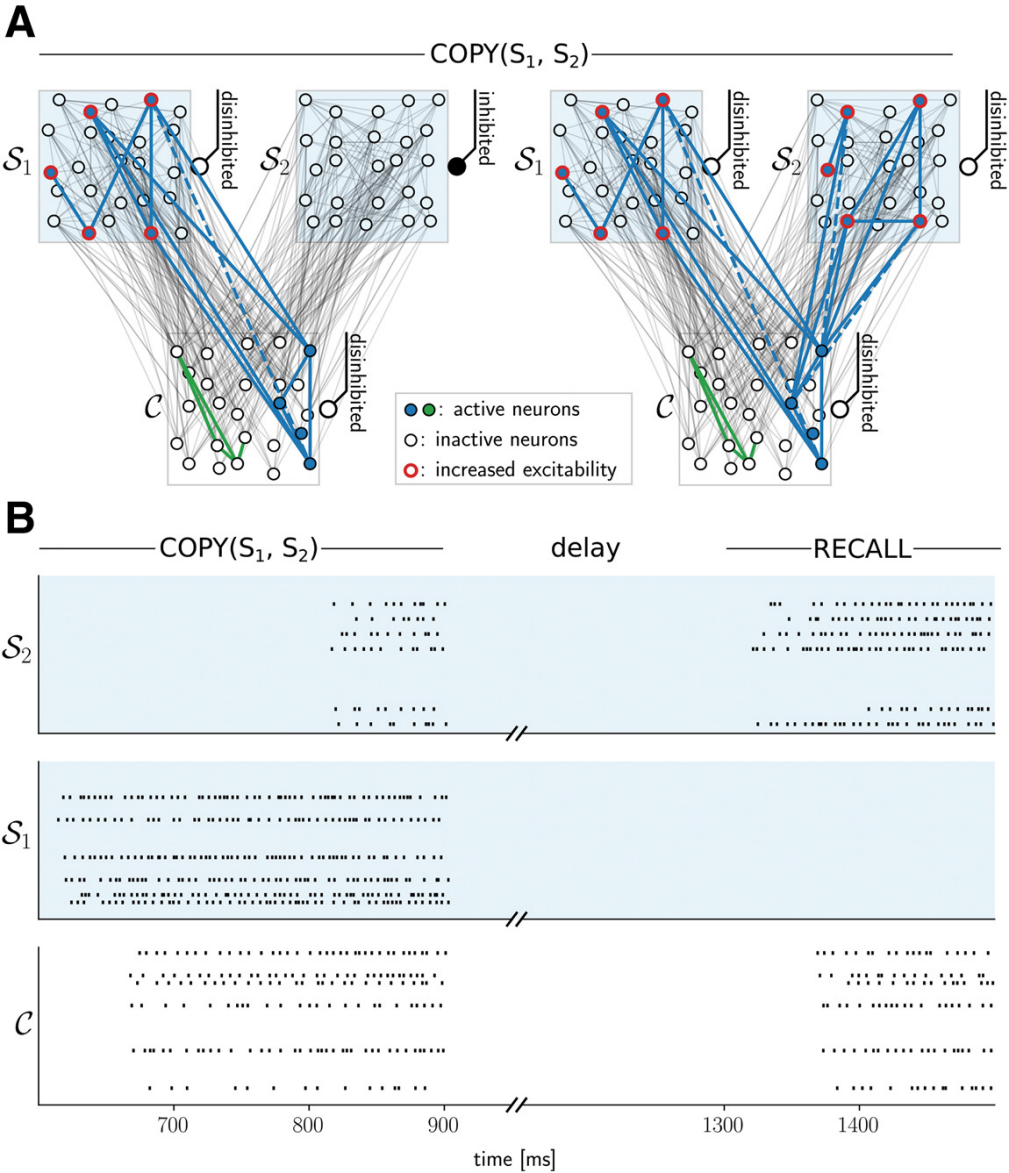
Assembly projection copy operation. ***A***, Copying information from one structural category to another through the creation of a new assembly projection (schematic drawing). Disinhibition of neural space $S_{1}$ recalls its content in content space C (left). A subsequent disinhibition of neural space $S_{2}$ creates an assembly projection for this content there (right). ***B***, Spike rasters from neural spaces $S_{2}$ (top), $S_{1}$ (middle), and content space C (bottom) in a simulated copy operation from $S_{1}$ to a $S_{2}$ (600–900 ms; only 20 randomly selected neurons are shown per space for clarity). After a 400-ms delay, the success of the copy operation is tested by performing a recall from neural space $S_{2}$ at time 1300 ms. The assembly is correctly recalled into the content space.

**Figure 6.**
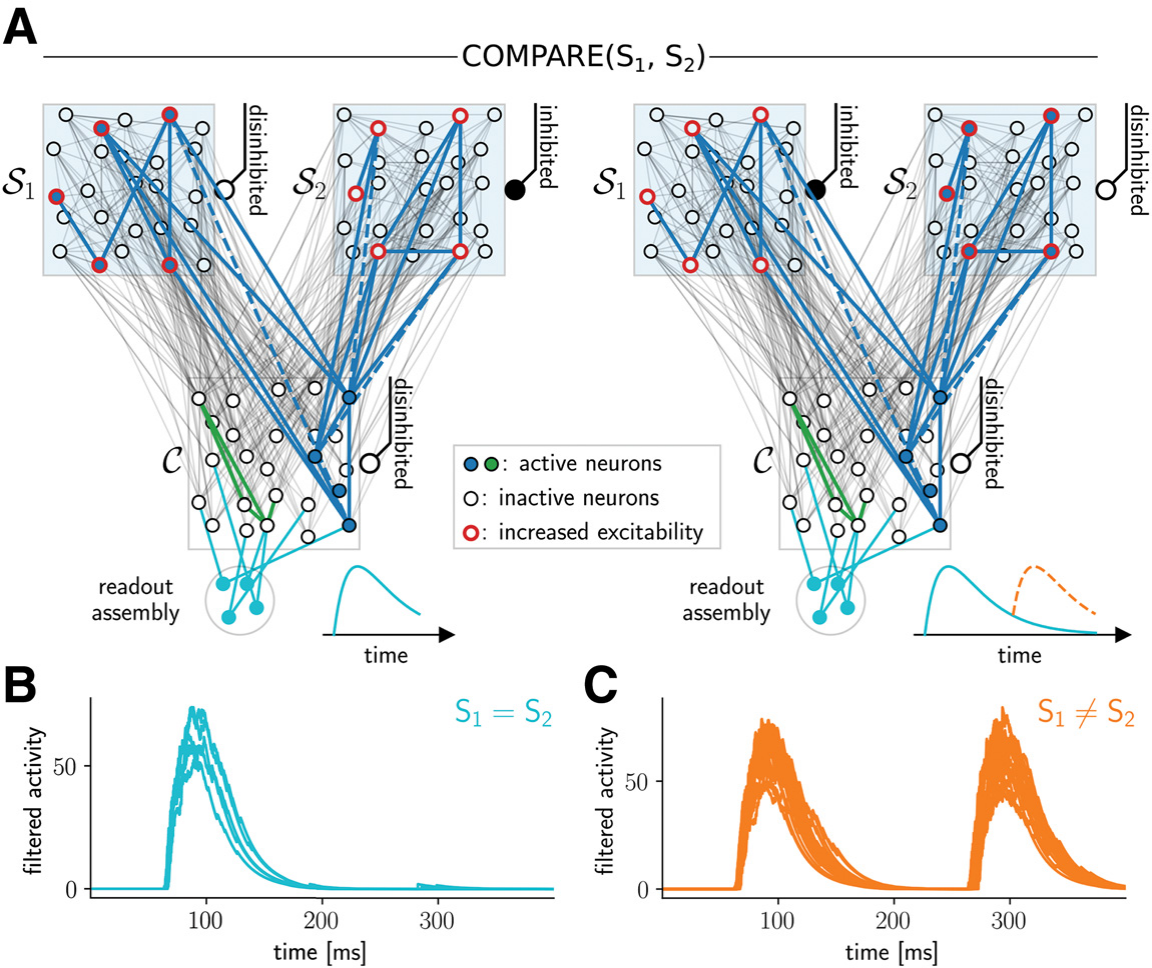
Comparison with assembly projections. ***A***, Comparing the content of two neural spaces (schematic drawing). A population of readout neurons (bottom, teal) receives sparse depressing connections from the excitatory neurons in the content space. The comparison consists of a recall from neural space $S_{1}$ (left; red neurons indicate neurons with higher excitability) followed by a recall from $S_{2}$ (right). During the first recall, readout weights become depressed and readout activity decreases (indicated by teal trace inset right of readout). Next, a second recall is performed (right). If the patterns are identical, the readout weights are still depressed and the readout response is therefore weak (teal trace at readout). If the content changes (i.e., $S_{2}\neq S_{1}$), readout weight from active neurons in C is not depressed, which leads to strong readout activity (dashed orange trace at readout). ***B***, ***C***, Resulting readout assembly activity in spiking neural network model. Each trace shows the population activity (filtered with an exponential kernel) of the readout population for one comparison operation between two assembly projection contents (25 comparisons in total, one for each possible way of assigning five values to two neural spaces $S_{1}$ and $S_{2}$). At time 0 ms, the content of neural space $S_{1}$ was recalled (during the first 50 ms of each recall, the content space remains inhibited and thus there is no readout activity), and the readout reacted in a similar manner to all contents. From time 200 ms on, the content of neural space $S_{2}$ was recalled. Because of depressed synaptic connections, the readout response was weak when the content of $S_{1}$ matched the content of $S_{2}$ (panel ***B***). In case $S_{1}$ and $S_{2}$ stored different values, the response was as strong as during the first recall phase (panel ***C***).

**Figure 7.**
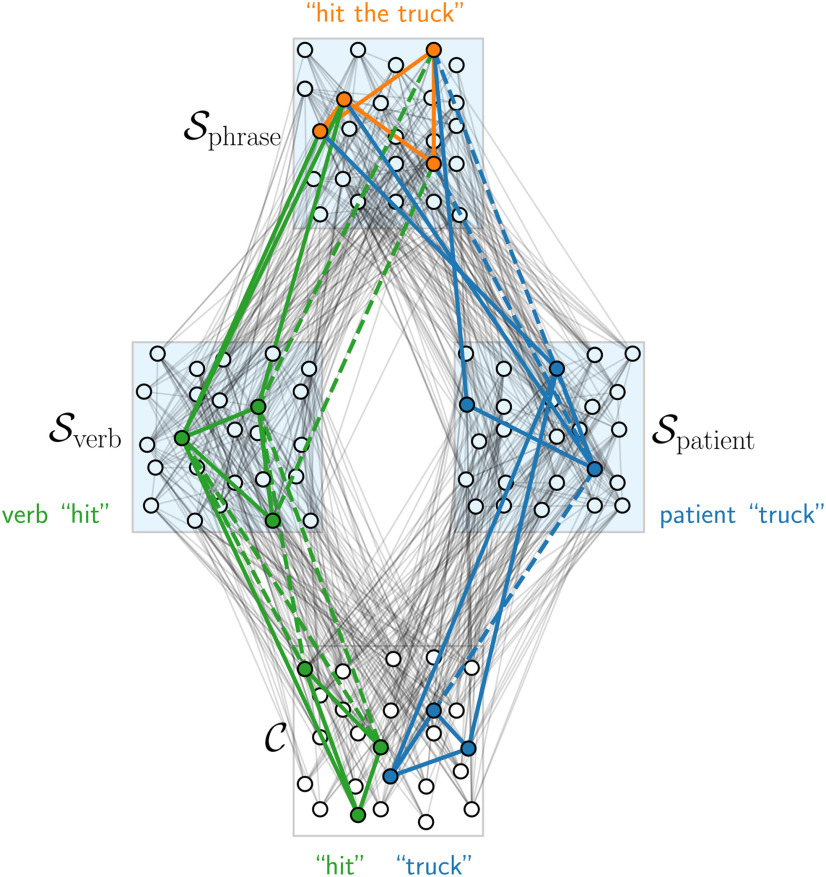
Extension of the assembly pointer model to multilevel pointers (conceptual figure). Conceptual architecture that supports a MERGE operation that merges two assembly representations in a neural space for verbs $S_{\text{verb}}$ and a neural space for patients $S_{\text{patient}}$ into a neural space for verb-patient phrases.

We show that structural information can be flexibly linked to content assemblies in this model and that the resulting activity of the network is consistent with the fMRI data by Frankland and Greene (2015). We further show that this model is capable of performing additional elementary processing operations proposed as atomic computational primitives in the brain (Marcus, 2003; Zylberberg et al., 2013; Marcus et al., 2014a): copying the content from one structural context to another, and the comparison of contents. Our results indicate that the concept of assembly projections extends the computational capabilities of neural networks in the direction of cognitive computation, symbolic computation, and the representation and computational use of abstract knowledge.

## Materials and Methods

### General network architecture

The network consists of one content space C as well as one or several neural spaces $S_{1},S_{2},$ . . . for structural information. Both the content space and each neural space consist of a pool of excitatory and a pool of inhibitory neurons (ratio, 4:1; the number of excitatory neurons is *N *=* *1000 for the content space and *N *=* *2000 for each neural space in our simulations). Excitatory and inhibitory neurons are sparsely interconnected. Within each space, excitatory neurons are connected by sparse recurrent connections with *p *=* *0.1 (i.e., for each pair of neurons, a connection between them is established with probability *p*, independently of all other pairs). Excitatory neurons in each neural space receive sparse excitatory connections (*p *=* *0.1) from the excitatory neurons in the content space C and vice versa. Since the connections are drawn at random, they are generally asymmetric. Neurons in C additionally receive input from an input population (*N*_in_ = 200 in our simulations).

### Connections between excitatory and inhibitory neurons

In the following, E and I denote the pool of excitatory and inhibitory neurons within the content space or a neural space, respectively. The parameters for these connections are based on data from mouse cortex (Avermann et al., 2012; Legenstein et al., 2017); the weights are static are given in Table 1.

**Table 1:** Connection parameters for static connections within each neural space and the content space

Connection	Probability	Synaptic weight	Synaptic delay
		pA	ms
E→I	0.575	17.39	0.5
I→E	0.6	–4.76	0.5
I→I	0.55	–16.67	0.5

Given are the parameters for connections between the excitatory population E

and the inhibitory population I as well as for recurrent connections from the inhibitory pool to itself. Recurrent excitatory connections are plastic and described in Table 2.

### Neuron model

We used a single neuron model for all neurons in our simulations. In this model, the probability of a spike of neuron *i* at time *t* is determined by its instantaneous firing rate

(1) $$\rho_{i}(t)=c_{1}\cdot V_{i}\prime (t)+c_{2}\cdot ({\text{e}}^{c_{3}\cdot V_{i}\prime (t)}-1)\;,$$

where *c*_1_, *c*_2_, and *c*_3_ can be used to achieve linear or exponential dynamics (we use *c*_1_ = 0, *c*_2_ = 1000 Hz, *c*_3_ = 1/mV for excitatory neurons and *c*_1_ = 10 Hz/mV and *c*_2_ = *c*_3_ = 0 for inhibitory neurons). $V_{i}\prime (t)$ is the effective membrane potential, which is calculated as $V_{i}\prime (t)=V_{i}(t)+b_{i,\text{sfa}}(t)$, where $b_{i,\text{sfa}}(t)$ is an adaptive bias which increases by some quantity *q*_sfa_ every time the neuron spikes (otherwise it decays exponentially with time constant *τ*_sfa_). $b_{i,\text{sfa}}(t)$ is also clipped at some value ${\hat{b}}_{\text{sfa}}$ (in our simulations, *q*_sfa_ = 0.02 mV, τ_sfa_ = 5 s and ${\hat{b}}_{\text{sfa}}$ = 0.5 mV for excitatory neurons in neural spaces; for all other neurons *q*_sfa_ = 0). The membrane potential is calculated using

(2) $$V_{i}(t)={\text{e}}^{-\Delta t/\tau_{\text{m}}}V_{i}(t-\Delta t)+(1-{\text{e}}^{-\Delta t/\tau_{\text{m}}})R_{\text{m}}\left(I_{i,\text{syn}}(t)+I_{i,\text{inh}}(t-\Delta t)+I_{\text{e}}\right),$$

where $\tau_{\text{m}}$ is the membrane time constant, $R_{\text{m}}$ is the membrane resistance, and $I_{\text{e}}$ is a bias current (τ_m_ = 10 ms, *R*_m_ = 0.5 MΩ; furthermore, *I*_e_ = 0.2 nA for excitatory and $I_{\text{e}}=0$ for inhibitory neurons). The current $I_{i,\text{syn}}(t)$ results from incoming spikes and is calculated via $I_{i,\text{syn}}(t)=\sum_{j}w_{ij}z_{j}(t)$, where *z_j_*(*t*) are incoming spikes, and *w_ij_* are weights assigned to the specific connection. $I_{i,\text{inh}}$ controls the disinhibition and is set to –4 nA for all neurons inside a neural space if it is inhibited, and 0 otherwise.

After a neuron has spiked, its membrane potential is reset to zero, and the neuron enters a refractory period. The duration of the refractory period (in milliseconds) is randomly chosen per neuron at the start of the simulation from a Γ distribution (*k *=* *4, *μ* = 3.5).

### Plastic connections

A simple model for spike timing-dependent plasticity (STDP) is used in the model for connections between excitatory neurons. Each pairing of a presynaptic and a postsynaptic spike with Δ*t* = *t*_post_ – *t*_pre_ leads to a weight change of

(3) $$\Delta w(\Delta t)=\{\begin{array}{ll} \eta \left({\text{e}}^{-\left|\Delta t\right|/\tau_{+}}-A_{-}\right) & \text{if }\Delta t\geq 0\\ \eta \alpha \left({\text{e}}^{-\left|\Delta t\right|/\tau_{-}}-A_{-}\right) & \text{if }\Delta t<0 \end{array},$$

where $\tau_{+},\tau_{-}>0$ are time constants determining the width of the learning window, $A_{-}$ determines the negative offset, *α* determines the shape of the depression term in relation to the facilitation term, and *η* is a learning rate. This rule is similar to the one proposed by Nessler et al. (2009), but without a weight dependency. The parameters for all plastic connections are given in Table 2. Weights were clipped between 0 and an upper bound dependent on the connection type (see Table 2). As we assume that disinhibition enables learning, the learning rates for all synapses within a neural space were set to zero during inhibition periods.

### Simulation

Network dynamics were simulated using NEST (Gewaltig and Diesmann, 2007; Eppler et al., 2008) with a time step of Δ*t *=* *0.1 ms (using PC hardware, Linux OS).

### Initial formation of assemblies in the content space

First, the content space learned to represent five very simple patterns presented by the 200 input neurons. Each pattern consisted of 25 active input neurons that produced Poisson spike trains at 100 Hz while other neurons remained silent (firing rate 0.1 Hz). Each input neuron was active in at most one pattern. This initial learning phase consisted of 200 pattern presentations, where each presentation lasted for 200 ms followed by 200 ms of random activity of the input neurons (all firing at 12.5 Hz to have a roughly constant mean firing rate of all input neurons). After the training phase, synaptic plasticity of connections between the input population and the content space as well as for recurrent connections within the content space was disabled.

After the training phase, each pattern was presented once to the content space for 200 ms, and the neuronal responses were recorded to investigate the emergence of assemblies. If a neuron fired with a rate of >50 Hz during the second half of this period, it was classified to belong to the assembly of the corresponding input pattern. This yields five assemblies in C; two of these are shown in Figure 1 (showing a subset of neurons of C, all large weights between neurons belonging to some assembly, i.e., >90% of the maximum weight, are shown with the color reflecting the assembly identity).

We created 10 instances of such content spaces (i.e., random parameters such as synaptic delays and recurrent connections were redrawn), which were separately trained.

### Creation of assembly projections (CREATE operation)

Depending on the experiment, one or two neural spaces were added ($S_{1}$ and $S_{2}$ ), each consisting of 2000 excitatory and 500 inhibitory neurons. Figure 1*A* shows the potential connections within the neural space S as well as potential feedforward and feedback connections (all existing connections denoted by thin gray lines). Stable assemblies were induced in each neural space separately by presenting each input pattern to the content space for 1000 ms (leading to the activation of the encoding assembly there; Fig. 1*B*), while one of the neural spaces was disinhibited (Fig. 1*C*). This was performed for every reported execution of an operation to obtain results which are independent of the specific network wiring.

Figure 1*C* shows emerging assemblies (measured as in the content space) in the neural space S during the CREATE phase for two different contents (all large recurrent, feedforward, and feedback connections involving the assembly in S, i.e., the active neurons, drawn in color; dashed lines denote feedback connections, i.e., from S to C).

In all following figures, assemblies and connectivity are plotted as in Figure 1.

When given in the text, statistics of weights for different connection types were obtained for a single content space instance (from the test set, see below) after five CREATE operations as previously described.

### Optimization of plasticity parameters

A total of 23 model parameters controlling synaptic plasticity (see Table 2) were optimized using a gradient-free optimization technique. All parameters were constrained to lie in a biologically reasonable range.

We used the RECALL operation (see Recall of content space assemblies) to assess the quality of parameters. The cost function penalized mismatches of neural activity in both neural spaces during the CREATE and RECALL periods. We defined the set $A_{\text{CREATE}}^{C}=\{n_{1},n_{2},...\}$ containing the neurons $n_{1},n_{2},...$, which were active in the content space C during the CREATE operation. Similarly, we defined $A_{\text{RECALL}}^{C}$ for the neurons active in C during the subsequent RECALL, as well as $A_{\text{CREATE}}^{S}$ and $A_{\text{RECALL}}^{S}$ for the neural space. The cost in some iteration was then given by

(4) $$C=\left|A_{\text{CREATE}}^{C}△A_{\text{RECALL}}^{C}\right|+\lambda \left|A_{\text{CREATE}}^{V}△A_{\text{RECALL}}^{V}\right|,$$

where A△B=(A∖B)∪(B∖A) is the symmetric difference between sets *A* and *B* and *λ* = 10^−4^ is a trade-off parameter.

The optimization procedure consisted of two steps: the initial parameter set was obtained by evaluating 230 candidates from a Latin Hypercube sampler (McKay et al., 1979) and choosing the parameter set with the lowest cost. Then, a gradient-free optimization technique using a fixed number of epochs (*N*_iter_ = 500) was used to further tune the parameters: in each iteration, a subset of parameters was chosen for modification (Bernoulli probability *p *=* *0.5 per parameter). For each selected parameter, a new value was drawn from a uniform distribution centered around the current value; the width of these proposal distributions was decayed linearly from half of the allowed parameter range (defined by the constraints as the difference between upper and lower bound for each parameter value) in the first iteration to 0.1% of the allowed range in the last. (The proposal distributions were also clipped to lie within the allowed range.) After evaluating the cost, the new parameter set was kept if the cost was lower than the cost of the previous parameter set, otherwise, the new parameters were discarded.

This optimization was performed using five pretrained content space instances: two were used for evaluating the costs and updating the parameters (cost terms were generated in two separate runs and summed to get the overall cost), three for early stopping (i.e., after the optimization, the parameter set giving the lowest cost when tested on these three content spaces was chosen as final parameter set). Using these parameters, the RECALL operation could reliably be performed on the five content space instances used during the optimization as well as on five new instances which were not used for optimization (i.e., the similarity criterion was met for each of the five values in each of the 10 content space instances; for details, see Results).

### Recall of content space assemblies (RECALL operation)

To test whether content can be retrieved from neural spaces reliably, we first presented a pattern to the network for 200 ms with one of the neural spaces $S_{1}$ or $S_{2}$ disinhibited. This corresponds to a brief CREATE operation. Note that because assemblies in the neural spaces were already created previously, the previously potentiated synapses were still strong. Hence, the shorter presentation period was sufficient to activate the assembly in the neural space. In the following, we refer to such a brief CREATE as a loading operation. After this loading phase, a delay period of 5 s followed (no input presented, i.e., input neurons firing at 12.5 Hz). In order to make sure that no memory was kept in the recurrent activity, all neural spaces were inhibited in this period. After the delay, we retrieved the content of the neural space in a RECALL operation, in which the neural space $S_{1}$ was disinhibited for 200 ms. During the first 50 ms of this phase, the content space remained inhibited.

We used a linear classifier to assess the success of such RECALL operations. The classifier was trained using the responses of neurons in C after assembly induction there (before the neural space was added to the circuit) by low-pass filtering the spike trains *S_i_*(*t*) of each neuron according to

(5) $$r_{i}(t)=\int_{0}^{T_{\text{LP}}}{\text{e}}^{-\frac{s}{\tau_{\text{LP}}}}S_{i}(t-s)ds,$$

with *τ*_LP_ = 20 ms and *T*_LP_ = 100 ms. The resulting vector of *r_i_* values (at a temporal resolution of 1 ms) was used as input to the classifier. We then used the trained classifier to classify the recalled content during the RECALL.

We also defined a similarity criterion based on the assembly overlap between the responses in C after training and during the RECALL. Neurons in the content space were classified as belonging to the assembly corresponding to the input pattern depending whether their firing rate was >50 Hz in the second half of the RECALL phase. We say that the similarity criterion is met if 80% of the neurons in the content space which belong to the assembly measured after assembly induction (see Initial formation of assemblies in the content space) are active during the RECALL phase while at the same time the number of excess neurons (active during RECALL but not after training) does not exceed 20% of the size of the original assembly.

### Robustness to parameter variations

We tested the robustness of the model by randomly varying its plasticity-controlling parameters and testing the performance of the RECALL operation. We randomly varied 20 of the parameters (Table 2, all highlighted parameters except for the *α* values controlling STDP shapes) by sampling each parameter value *v* from a normal distribution with mean *v* and SD $\sigma_{\text{param}}v$. Some clipping was necessary after sampling (time constants were forced to be non-negative, weight initial values were clipped so that the higher bound of the uniform distribution was larger than the lower bound). We generated 100 different random parameter combinations this way for a number of values of *σ*_param._ As *σ*_param_ increases, the parameters deviate more strongly from their initial values. Still, the readout error for the RECALL operation remained quite low even for substantial changes of the parameter values (e.g., for $\sigma_{\text{param}}=0.2$, the mean readout error was <1%). We also evaluated the similarity criterion defined in section Recall through assembly projections (see Results, Emergence of assembly projections through Hebbian plasticity) that was based on the similarity of recalled assemblies to input-driven assemblies. The percentage of trials when this criterion is met is very high even for substantial parameter variations.

We furthermore tested the robustness to a few model parameters in particular. In all reported simulations, we induced assemblies in content space C by 200 presentations of each input pattern. When varying the number of presentations, we found that assemblies in content space were also robustly induced with only 25 presentations (for all input patterns, i.e., each individual pattern was presented five times on average). When testing the RECALL operation after such reduced induction, the mean readout error remained below 1%. We next asked whether the firing rates of input neurons had a significant impact on recall performance. In this second test, we reduced the firing rates of active input neurons from 100 to 60 Hz while increasing the rate of inactive neurons from 0.1 to 1 Hz. Again, the mean readout error remained below 1% when testing the RECALL operation. In our standard model, both the content space and the neural spaces are spontaneously active (5.5 and 2.6 Hz, respectively). Spontaneous activity is a hallmark of cortical circuits. In our model, it arises from the chosen model for interacting excitatory and inhibitory populations (Jonke et al., 2017; Legenstein et al., 2017). In a third test, we asked whether these specific baseline firing rates were crucial for the model. We therefore changed the parameters so both *C* and S had either low (≈2.7 Hz) or high (≈5.5 Hz) base firing rates in the absence of input. We found that in both cases, the mean readout error at RECALL remained below 1%.

Finally, we tested the capacity of the model with respect to the number of concepts which can be stored in C and stored/recalled in/from S. We found that increasing the number of concepts reduced the assembly size per concept. When encoding 30 concepts in C, concept assemblies consisted of only (7–29 neurons). Nevertheless, RECALL still worked reasonably well (mean readout error 2.5%). When further increasing the number of concepts in C, RECALL performance collapsed (35 concepts: mean readout error 11.3%, 40 concepts: 46.1%). We have chosen for this study a rather small network, and we were more interested in general functional questions than in questions of capacity. Network capacity has been rigorously analyzed for Hopfield-type networks (Amit et al., 1985). According to these analyses, it is expected that network capacity should increase linearly with the number of neurons in the network. While storage capacity in the content space should in principle follow these classical analyses, recall performance is an interesting open question that needs further investigations.

### Details to the attachment of roles to words

These experiments modeled the findings in (Frankland and Greene, 2015) regarding the attachment of roles to words in temporal cortex. We again used the network described above with one content space C and two neural spaces for structural information, which we refer to in the following as $S_{\text{agent}}$ and $S_{\text{patient}}$. Input patterns to the network were interpreted as words in sentences. We used the network described above with five assemblies in C that represented five items (words) $A_{1},...,A_{5}$ and five assembly projections in each neural space (created as before). We defined that *A*_1_ represents “truck” and *A*_2_ represents “ball.” We considered the four sequences, corresponding to four sentences: S1 = 〈agent = “truck,” patient = “ball”〉, S2 = 〈patient = “ball,” agent = “truck”〉, S3 = 〈patient = “truck,” agent = “ball”〉, S4 = 〈agent = “ball,” patient = “truck”〉. The processing of a sentence was modeled as follows. The words “truck” and “ball” were presented to the network (i.e., the corresponding input patterns) in the order of appearance in the sentence, each for 200 ms, without a pause in between. During the presentation of a word, an assembly projection with the active assembly in C was created in $S_{\text{agent}}$ if it served the role of the agent or alternatively in $S_{\text{patient}}$ if its role was the patient. For example, for the sentence “The truck hit the ball,” the input “truck” was presented, and a projection was formed with $S_{\text{agent}}$, then “ball” was presented and a projection was formed with $S_{\text{patient}}$. The sequence of sentences S1 to S4 was presented twice to the network. The classifier described in the following was trained on the first sequence and tested on the second sequence.

Spiking activity was recorded in all spaces. The first 50 ms of each word display were discarded to allow the activity to settle. Spikes were then low-pass filtered (as above; Eq. 5) to obtain the filtered activity *r_i_*(*t*) for each neuron *i*. To emulate low-dimensional fMRI signals, we randomly divided the neurons in each space into five groups (“voxels”). The filtered activities were summed over neurons (i.e., over *i*) within each voxel and averaged over time (i.e., over *t*), resulting in a coarse signal. We additionally added independent Gaussian noise (mean: 0, variance: 5) to each datapoint. We denote by $r_{\text{agent}},\;r_{\text{patient}},\;r_{S}$, and $r_{C}$ the low-dimensional noisy signal vector from neural space $S_{\text{agent}}$, from neural space $S_{\text{patient}}$, from both neural spaces (concatenated), and from the content space, respectively.

The task for the first classifier was to classify the meaning of the current sentence for each presentation (this is equivalent to determining the role of the truck in the discussed example). Hence, the sentences S1 and S2 constituted class *C*_0_ and sentences S3 and S4 the class *C*_1_. The classification was based on the network activity $r_{S}$ from the neural spaces. Using Scikit-learn (version 0.19; Pedregosa et al., 2011), we trained a classifier for logistic regression using the traces from the neural spaces. For comparison, a classifier was also trained in the same manner on network activity $r_{C}$ from the content space.

To model the second experiment from (Frankland and Greene, 2015), we considered sentences that were formed by tuples from the set of all five items $A_{1},...,A_{5}$ (see Results). Then, the task for a second classifier (“who is the agent”) was to use the low-dimensional network activity from the neural space for the agent $r_{\text{agent}}$ to classify the identity of the current agent during those times when $S_{\text{agent}}$ was disinhibited. The activity traces were created as in the previous experiment. The data set was divided into a training set and a test set as described in Results, and a classifier was trained (as above, “agent decoder”). Finally, the task for a third classifier (“patient decoder”) was to classify from the subsampled network activity of the neural space for the patient $r_{\text{patient}}$ the identity of the current patient during those times when $S_{\text{patient}}$ was disinhibited. The procedure was analogous to the procedure for the second classifier.

### Details to copying of assembly projections (COPY operation)

We tested the COPY operation using simulations with one content space and two neural spaces. After a content was loaded into $S_{1}$ and a brief delay period (400 ms), a RECALL operation was performed from $S_{1}$ (duration 200 ms as above). Then, $S_{2}$ was additionally disinhibited for 100 ms. To test the performance, a RECALL was initiated from $S_{2}$ 400 ms later (same similarity criterion as above). We report the results on the same five network instances as before.

### Details to comparison of assembly projections (COMPARE operation)

The simulations testing the COMPARE operation again used one content space and two neural spaces. We tested 25 comparisons in total, one for each possibility how two neural spaces $S_{1}$ and $S_{2}$ can form assembly projections with the five contents represented by the content space C. The readout assembly consisted of 50 integrate-and-fire neurons (resting potential –60 mV, firing threshold –20 mV, membrane time constant 20 ms, refractory period of 5 ms with reset to the membrane potential) with sparse incoming connections from excitatory neurons in the content space (connection probability 0.1) with depressing synapses (Markram et al., 1998; parameters taken from Gupta et al. (2000), type F2; connection weight 50 pA). After two load operations (duration 200 ms, each followed by 50 ms of random input), which store two assembly projections (to identical or different contents in C ) in the neural spaces $S_{1}$ and $S_{2}$, we performed a recall operation from each (400 ms in total, no delay). During this time, the spike trains *S_i_*(*t*) for neuron i from the readout assembly were recorded and filtered (as above; Eq. 5) to obtain the low-pass filtered activity for each neuron *i* (*T*_LP_ = 100 ms, *τ*_LP_ = 20 ms). We calculated the activity of a readout population as $R_{\text{readout}}(t)=\sum_{i}r_{i}(t)$. 

### Code accessibility

The code used to produce the results in the paper is freely available online at https://github.com/IGITUGraz/structured_information_representation. The code is available as Extended Data 1.

10.1523/ENEURO.0533-19.2020.ed1 Extended Data 1 Source code for computer simulations. Download Extended Data 1, ZIP file.

## Results

### Generic network model for assembly projections

Experimental data obtained by simultaneous recordings from large sets of neurons through multielectrode arrays or Ca^2+^ imaging showed that neural activity patterns in cortex can be characterized in first approximation as spontaneous and stimulus-evoked switching between the activations of different (but somewhat overlapping) subsets of neurons (Buzsáki, 2010; Bathellier et al., 2012; Luczak and MacLean, 2012). These subsets of neurons are often referred to as neuronal assemblies. Consistent with this experimental data, both specific content experienced through sensory inputs and more abstract structural information are represented in our model by corresponding assemblies of neurons in generic neural circuits. In particular, we assume that content, which could be words, concepts, values, etc., is encoded by assemblies in a content space C (Fig. 1*A*, bottom).

We assume that there exist specific cortical subareas for some abstract structural categories. We model such subareas for abstract categories $S_{1},S_{2},...$ as a distinct set of neural spaces $S_{1},S_{2},...$. A single neural space is indicated in Figure 1*A*, top. Each neural space can be viewed as functioning like a register in a computer. But in contrast to such registers, neural spaces for structural information do not store content directly. Instead, we hypothesize that a storage operation leads to the emergence of an assembly in the neural space. This assembly will preferentially include neurons which are linked (by chance) via sufficiently strong synapses to the assembly representing the particular content in the content space. The synaptic connections between these assemblies will further be strengthened (in both directions) by synaptic plasticity. We call these projections of assemblies to neural spaces assembly projections. In other words, by assembly projection, we mean the emergence of an assembly in a neural space as the result of afferent activity by an assembly in the content space, with the establishment of strong synaptic links between them.

Importantly, we do not assume specifically designed neural circuits which enable the creation of such assembly projections. Instead, we assume a rather generic network for the content space and each neural space, with lateral excitatory connections and lateral inhibition within the space (a common cortical motif, investigated by Jonke et al., 2017; Legenstein et al., 2017). Furthermore, we assume that neurons in the content space are sparsely connected to neurons in neural spaces and vice versa (Fig. 1*A*). We will show that the creation of an assembly projection in a neural space, implementing the attachment of structural information to some content, emerges naturally in such generic circuits with random connectivity from plasticity processes.

In addition, our model takes into account that neurons typically do not fire just because they receive sufficiently strong excitatory input. Experimental data suggest that neurons are typically prevented from firing by an “inhibitory lock” which balances or even dominates excitatory input (Haider et al., 2013). Thus, a generic pyramidal cell is likely to fire because two events take place: its inhibitory lock is temporarily lifted (“disinhibition”), and its excitatory input is sufficiently strong. A special type of inhibitory neuron (VIP cells) has been identified as a likely candidate for triggering disinhibition, since VIP cells target primarily other types of inhibitory neurons (PV+ and SOM+ cells) that inhibit pyramidal cells (Harris and Shepherd, 2015). Firing of VIP cells is apparently often caused by top-down inputs (VIP cells are especially frequent in layer 1, where top-down and lateral distal inputs arrive). Their activation is conjectured to enable neural firing and plasticity within specific patches of the brain through disinhibition (Pfeffer, 2014; Caroni, 2015; Froemke and Schreiner, 2015; Fu et al., 2015; Letzkus et al., 2015). One recent study also demonstrated that long-term plasticity in the human brain can be enhanced through disinhibition (Cash et al., 2016). We propose that top-down disinhibitory control plays a central role for neural computation and learning in cortex by initiating, for example, the creation and reactivation of assembly projections. We note that we are not investigating the important question: which neural processes resulted in the decision to disinhibit this particular neural space, that is, to decide whether a specific structural information is attached to the current content. We modeled disinhibition of neural and content spaces in the following way. As a default, excitatory neurons received an additional strong inhibitory current that silenced the neural space. When the space was disinhibited, this inhibitory current was removed, which enabled activity in the neural space if excitatory input was sufficiently strong (see Materials and Methods).

### Emergence of assembly projections through Hebbian plasticity

To test whether the assignment of structural information to content can be performed by generic neural circuits, we performed computer simulations where stochastically spiking neurons (for details, see Materials and Methods) were embedded in the following network structure (Fig. 1*A*): the network consisted of a content space C with 1000 excitatory neurons and a single neural space S for structural information consisting of 2000 excitatory neurons. In both spaces, neurons were recurrently connected (connection probability 0.1), and lateral inhibition was implemented by means of a distinct inhibitory population to ensure sparse activity (excitatory to inhibitory neuron ratio, 4:1; connectivity between excitatory and inhibitory neurons was based on data; Avermann et al., 2012; see Materials and Methods; Table 1). Because of stochastic spiking of network neurons, both C and any neural space S exhibited spontaneous activity (mean firing rate for disinhibited C and S in the absence of input: 5.5 and 2.6 Hz, respectively; specific spontaneous rate values do not seem too important, see Materials and Methods). Connections between C and S were introduced randomly with a connection probability of 0.1. Neurons in the content space additionally received connections from 200 input neurons whose activity indicated the presence of a particular input stimulus.

Hebbian-type plasticity is well known to stabilize assemblies (Litwin-Kumar and Doiron, 2014; Pokorny et al., 2020), and it can also strengthen connections bidirectionally between neural spaces and the content space. In our model, this plasticity has to be reasonably fast so that connections can be strengthened within relatively short stimulus presentations. As a Hebbian-type plasticity, we used in our spiking neural network model STDP (Bi and Poo, 1998; Caporale and Dan, 2008) for all synapses between excitatory neurons in the circuit (for more details, see Materials and Methods). Other synapses were not plastic.

We will in the following describe the behavior of this generic neural network model, how assembly projections emerge, and how structured information can be recalled. We will use the running example of nouns in sentences as content and their semantic role as the structural category. However, we emphasize that the model is not restricted to semantic roles or language processing, it rather is a model for the general enrichment of content with structural categories.

#### Emergence of content assemblies

We do not model the initial processing of sensory input, which is in itself a complicated process. Instead, we assume that assemblies in content space act as tokens for frequently observed input patterns that have already been extracted from the sensory stimulus. Hence, we induced assemblies in content space C by an initial repeated presentation of simple rate patterns provided by 200 spiking input neurons (see Materials and Methods). We first defined five such patterns $P_{1},,P_{5}$ that modeled the input to this space when a given content (e.g., the word “truck” or “ball”) is experienced. These patterns were repeatedly presented as input to the disinhibited content space (the value space remained inhibited during this presentation). Because of these pattern presentations, an assembly $C(P_{i})$ emerged in content space for each of the patterns *P_i_* (assembly sizes typically between 50 and 90 neurons) that was robustly activated (average firing activity of assembly neurons >50 Hz) whenever the corresponding pattern was presented as input (Fig. 1*B*). STDP of recurrent connections led to a strengthening of synapses within each assembly (mean weight ± SD: 0.59 ± 0.01 pA), while synapse weights between assemblies were depressed (0.00 ± 0.001 pA; for details, see Materials and Methods).

#### Emergence of assembly projections

Assume that an active assembly C(P) in content space represents some content *P* (such as the word “truck”). A central hypothesis of this article is that disinhibition of a neural space S leads to the creation of an assembly projection S(P) in S. This projection S(P) is itself an assembly (like the assemblies in the content space) and interlinked with C(P) through strengthened synaptic connections.

To test this hypothesis, we next simulated disinhibition of the neural space S while input to content space C excited an assembly there. This disinhibition allowed spiking activity of some of the neurons in S, especially those that received sufficiently strong excitatory input from the currently active assembly in the content space. STDP at the synapses that connected the content space C and the neural space S led to the stable emergence of an assembly $S(P_{i})$ in the neural space within one second when some content *P_i_* was represented in C during disinhibition of S (Fig. 1*C*). The emerging assemblies in the neural space had strong synaptic ties to the representation of *P_i_* in C in both feedforward (weights: 1.25 ± 0.001 pA with the assembly representing *P_i_* vs 0.54 ± 0.11 pA with assemblies representing other contents) and feedback (0.86 ± 0.03 pA with *P_i_* vs 0.00 ± 0.02 pA with others) directions. Further, plasticity at recurrent synapses in S induced strengthening of recurrent connections within assemblies there (weights within assemblies: 1.09 ± 0.04 pA, weights between assemblies: 0.10 ± 0.14 pA; Fig. 1*C*; Materials and Methods). Hence, disinhibition led to the rapid and stable creation of an assembly in the neural space S, i.e., an assembly projection. We denote the attachment of structural information S (by an assembly projection in S ) to content *P* by CREATE(S,P). 

Fast recruitment of assemblies in a neural space necessitates rapid forms of plasticity. We assumed that a (possibly initially transient) plasticity of synapses occurs instantaneously, even within seconds. The assumption of rapid plasticity of neurons and/or synapses is usually not included in neural network models, but it is supported by a number of recent experimental data. In particular, Ison et al. (2015) showed that neurons in higher areas of the human brain change their response to visual stimuli after few or even a single presentation of a new stimulus where two familiar images are composed into a single visual scene.

#### Recall through assembly projections

From a functional perspective, the structural information that has been attached to some content can be exploited at a later point by recalling the content linked to this structural category. In our model, a recall RECALL(S) for a category S should lead to the activation of the assembly C(P) in content space which was active at the most recent CREATE(S,P) operation. The strengthened synaptic connections between assemblies in neural space S for structural category S and content space C may in fact enable such a recall. However, an additional mechanism is necessary that reactivates the most recently active assembly in neural space S. One possible candidate mechanism is the activity-dependent change of excitability in pyramidal cells. It has been shown that the excitability of pyramidal cells can be changed in a very fast but transient manner through fast depression of GABAergic synapses onto pyramidal cells (Kullmann et al., 2012). This effect is potentially related to the match enhancement or match suppression effect observed in neural recordings from monkeys, and is commonly used in neural network models for delayed match-to-sample (DMS) tasks (Tartaglia et al., 2015). Using such a mechanism, a RECALL(S) can be initiated by disinhibition of the neural space S while the content space does not receive any bottom up input (Fig. 2*A*). The increased excitability of recently activated neurons in S ensures that the most recently active assembly is activated which in turn activates the corresponding content through its (previously potentiated) feedback connections to content space C. 

We tested whether such a mechanism can reliably recall previously bound contents from structural categories in our generic network model. A transient increase in neuron excitability has been included in the stochastic spiking neuron model through an adaptive neuron-specific bias that increases slightly for each postsynaptic spike and decays with a time constant of 5 s (see Materials and Methods). We used an automated optimization procedure to search for synaptic plasticity parameters that lead to clean recalls (see Materials and Methods; all parameters were constrained to lie in biologically realistic ranges). Figure 2*B* shows the spiking activity in our spiking neural network model for an example recall 5 s after the creation of the assembly projection. One sees that the assembly pattern that was active at the create operation was retrieved at the recall.

In general, we found that the contents of the projection can reliably be recalled in our model. In order to test whether the model can deal with the high variability of individual randomly created networks as well as with the variability in sizes and connectivity of assemblies, we performed several simulations with random network initializations. We used five different content space instances with randomly chosen recurrent connections. In each of those, we induced five stable assemblies encoding different contents as described above. Note that these assemblies emerged through a plasticity process, so their sizes were variable. For each of these five content space instances, we performed 10 simulations where the neural space was set up and randomly connected in each of these. This procedure ensured that we did not test the network behavior on a particular instantiation of the circuit, but rather whether the principle works reliably for generic randomly connected circuits.

To quantify the quality of a RECALL operation, we assessed whether a linear readout could identify the recalled pattern from the activity in the content space C. The readout was first trained before any CREATE operation, i.e., before an assembly projection in a neural space was established. We activated each assembly in C and trained the linear classifier on the low-pass filtered spike trains of the neurons in C (for details, see Materials and Methods). During a RECALL, we then asked whether the readout could correctly classify the recalled content from activity in content space at any point in time 50 ms after the start of the RECALL. We found that the classification error of the readout was 0.9 ± 0.2% (mean ± SD) over 250 trials (five content spaces with five patterns each, and this repeated 10 times for different neural spaces). We hypothesized that this high classification accuracy was possible due to a strong similarity between the input-driven assembly activity and the activated assembly at the RECALL in content space. To test this hypothesis, we measured the overlap of the assembly encoding a concept in C directly after this assembly has been established there and the neurons active in C during the RECALL. We define a similarity criterion as follows: we say that a recall meets the similarity criterion if during the RECALL phase, at least 80% of the neurons that were active after assembly induction in content space are also active (firing rate > 50 Hz) during recall, and if the number of erroneously active neurons does not exceed 20% of the original assembly size. All 250 tested RECALL operations met this criterion. In a typical run, one or two neurons from the original assembly were missing (mean ± SD: 2.1 ± 1.6), but no excess neurons fired during the RECALL (0.1 ± 0.5).

These results show that content can be successfully recalled from established assembly projections in a rather generic network model. In order to test the robustness of the model to parameter variations, we performed additional simulations where parameters were varied randomly (Fig. 3*A*,*B*). We found that the model can reliably recall content even under large random variations of the parameters. In addition, we found that these results were robust to changes in input firing rates, spontaneous firing rates of the recurrent networks, the number of initial pattern presentations, and the number of stored concepts (for details, see Materials and Methods, Robustness to parameter variations).

### Reproducing experimental data on the attachment of roles to words and structured information representation

Two experiments performed by Frankland and Greene (2015) provided new insights in how structural information may be attached to words in neocortex. Sentences were shown to participants where individual words (like “truck” or “ball”) occur as the agent or as the patient. The authors then studied how cortex represents the information contained in a sentence. In a first experiment, the authors aimed to identify cortical regions that encode sentence meaning. Example sentences with the words “truck” and “ball” are “The truck hit the ball” and “The ball hit the truck.” The different semantics of these sentences can be distinguished, for example, by answering the question “What was the role of the truck?” (with the answer “agent” or “patient”). Indeed, the authors showed that a linear classifier is able to distinguish sentences meaning from the fMRI signal of lmSTC. Using our model for assembly projections, we can model such situations by attaching to words either the structural category agent (“who did it”) or the structural category patient (“to whom it was done”). Under the assumption that lmSTC hosts neural spaces (with assembly projections) for the role of words in a sentence, it is expected that decoding of the word’s role is possible from the activity there, but not from the activity in content space where the identities are encoded independently of their role. We performed simulations where we sequentially presented input representing the words “truck” and “ball” to the content space, which activated corresponding assemblies there. Here, the temporal order was mimicking the position of the word in the sentence (e.g., in “The truck hit the ball” and “The ball hit the truck”). During presentation, assembly projections were created either to neural space $S_{\text{agent}}$ or $S_{\text{patient}}$, depending on the roles of the words. Note that we did not model the processing of the verb or other words in the sentence, as only the representation of the agent and the patient was investigated by Frankland and Greene (2015). We recorded activity from the neural spaces. To mimic fMRI conditions, we severely reduced spatial and temporal resolution by summing activity of neurons within each of five randomly chosen equally sized groups (representing “voxels”) as well as averaging activity temporally over the whole time of the concept presentation. We then added noise to these five-dimensional signals (see Materials and Methods). We found that a linear classifier trained on these signals was able to perfectly classify sentences from a held-out test set (error 0% over five content space instances) when using the signals from the neural spaces. On the other hand, a classifier based on activity of the content space performed only slightly better than random with a test classification error of 47.0%.

A second experiment by Frankland and Greene (2015) revealed that information from lmSTC subregions can also be used to read out the word that is the agent (or patient) in the current sentence. More specifically, the authors showed that it is possible to predict the identity of the agent from the fMRI signal of one subregion of lmSTC and the identity of the patient from the signal in another subregion (generalizing over all identities of other roles and over different verbs). We expected that this would also be the case in the proposed model since the assemblies that are formed in the neural spaces $S_{\text{agent}}$ and $S_{\text{patient}}$ are typically specific to the linked content. We tested this hypothesis by training a multinomial logistic regression model to classify the linked content for each of the two neural spaces (agent and patient) at times when these spaces were disinhibited (Fig. 4, “agent decoder” and “patient decoder”). Here, we created assembly projections for words as before, but we considered all 40 possibilities of how five items (words) $A_{1},...,A_{5}$ (white, bottom) and neural space S can be sequentially linked (for example, $S_{\text{agent}}$ is attached first to *A*_1_, then $S_{\text{patient}}$ is attached to *A*_2_; we excluded sentences where the agent and patient is the same word). Low-pass filtered activity of a subset of neurons was sampled at every 1 ms to obtain the feature vectors to the classifiers (see Materials and Methods). Half of the possible sequences were used for testing where we made sure that the two items used in a given test sentence have never been shown in any combination in one of the sentences used for training. Consistent with the results of Frankland and Greene (2015), the classifier achieved optimal classification performance on test data (classification error <0% for each neural space). Note that such classification would fail if each neural space consisted of only a single assembly that is activated for all possible contents (Zylberberg et al., 2013), since in this case no information about the identity of the role is available in the neural space (see Discussion).

### Cognitive computations with assembly projections

Apart from the creation of assembly projections and recall of content, the implementation of basic operations on symbols have been postulated to be essential for many higher cognitive functions (Marcus, 2003). Two such operations have been considered by Zylberberg et al. (2013). The first is $\text{COPY}(S_{1},S_{2})$, which copies (or routes) the information attached to one structural category to some other category. Copying of information is trivial in digital computers, where bits strings can simply be moved between registers or memory. In neuronal networks of the brain however, the common assumption is that content is represented by currently active assemblies, which cannot be moved to other brain regions. In our model, the copy operation can be realized by creation of an assembly projection in neural space $S_{2}$ to the content which the assembly projection in neural space $S_{1}$ refers to. We hypothesized that this operation can be implemented in our generic circuit model simply by disinhibiting $S_{1}$ to activate the corresponding content in C followed by a disinhibition of $S_{2}$ to create an assembly projection there (Fig. 5*A*).

To test this hypothesis, we performed simulations of our spiking neural network model with one content space and two variable spaces. The performance was tested through a recall from the target assembly projection 400 ms after the projection was copied (Fig. 5*B*). We deployed the same setup as described above where five assemblies were established in the content space, again considering five different pretrained content space instances (see above). For each of these, we performed 10 copy operations (testing twice the copying of each content assembly) and assessed the assembly active in the content space after a recall from the target variable space. Again, the readout error was negligible (0.9 ± 0.6%), and all of the 50 considered cases met the similarity criterion as defined above.

A final fundamental operation considered by Zylberberg et al. (2013) is $\text{COMPARE}(S_{1},S_{2})$, which assesses whether the content attached $S_{1}$ is equal to the content attached to $S_{2}$. One possible implementation of this operation in our model is established by a group of readout neurons which receive depressing synaptic connections from the content space. Then, when the contents of $S_{1}$ and $S_{2}$ are recalled in sequence, the readout synapses will be depressed for the content of $S_{2}$ if and only if the content of $S_{2}$ equals the content of $S_{1}$. Such a “change detecting” readout population thus exhibits high activity if the contents of $S_{1}$ and $S_{2}$ are different (Fig. 6*A*). Simulation results from our spiking neural network model are shown in Figure 6*B*,*C*. Using a set of five contents as above, we tested 25 comparisons in total, one for each possibility how these five contents can be attached to two neural spaces $S_{1}$ and $S_{2}$. Figure 6*B* shows readout activity for the case when the same content was stored by both $S_{1}$ and $S_{2}$ (five cases). The readout activity of the second recall (starting after time 200 ms) was virtually absent in this case. In contrast, if the different contents were stored (20 cases), the second recall always induced strong activity in the readout population (Fig. 6*C*). Hence, this simple mechanism is sufficient to compare contents attached to structural categories with excellent precision by simply thresholding the activity of the readout population after the recall from the second assembly projection.

## Discussion

It has often been emphasized (Marcus, 2003; Marcus et al., 2014a; Behrens et al., 2018) that there is a need to understand brain mechanisms for information processing via factorized structured representations and variable-like mechanisms. We show in this article how structured information representation and processing can be performed in a generic network of spiking neurons by means of assembly projections. Our model is consistent with recent findings on cortical assemblies and the encoding of sentence meaning in cortex (Frankland and Greene, 2015). Our neural network model is not specifically constructed to perform such tasks. Instead, it is based on generic sparsely and randomly connected neural spaces that organize their computation based on fast plasticity mechanisms. The model provides a direct link between information processing on the algorithmic level of symbols and sentences and processes on the implementation level of neurons and synapses. The resulting model for brain computation supports top-down structuring of incoming information, thereby laying the foundation of goal oriented “willful” information processing rather than just input-driven processing. The proposed synaptic plasticity that links assemblies in neural spaces with content representations can be transient, but could also become more permanent if its relevance is underlined through repetition and consolidation. This would mean that some neurons in the neural space are no longer available to form new projection assemblies, but this does not pose a problem if each neural space is sufficiently large.

### Related work

A large body of modeling studies have tackled related problems in the context of the general binding problem. We study in this work not the standard form of a binding task, where several features are bound together to constitute an object representation (Treisman, 1996). Instead, we are addressing the problem of binding abstract categories, i.e., structural information, to content. This is more closely related to variable binding. In the example of language processing alluded to above, each neural space can in this view also be interpreted to represent one variable (one for “agent” and one for “patient”) that is bound to the content (the word in the sentence). The main classes of models in this direction are pointer-based models, models based on indirect addressing, anatomic binding models, neural blackboard architectures, and vector symbolic architectures. We discuss the relation of our model to these proposals in the following. Pointer-based models (Zylberberg et al., 2013) assume that pointers are implemented by single neurons or co-active populations of neurons which are synaptically linked to content. In contrast, our model is based on the assumption that distributed assemblies of neurons are the fundamental tokens for encoding symbols and content in the brain, and also for projections which implement in our model some form of pointer. We propose that these assembly projections can be created on the fly in some neural spaces and occupy only a sparse subset of neurons in these spaces. Frankland and Greene (2015) showed that the identity of a thematic role (e.g., the agent in a sentence) can be predicted from the fMRI signal of a subregion in temporal cortex when a person reads a sentence. As shown above, this finding is consistent with assembly projections. It is, however, inconsistent with models where a variable engages a population of neurons that is independent of the bound content, such as pointer-based models. In comparison to pointer models, the assembly projection model could also give rise to a number of functional advantages. In a neural space S for some variable *S*, several instantiations of the variable can coexist at the same time, since they can be represented there by increased excitabilities of different assemblies. These contents could be recalled as different possibilities in a structured recall and combined in content space C with the content of other variables to answer more complex questions.

Another class of models is based on the idea of indirect addressing. These models assume that a neural space for a variable encodes an address to another brain region where the corresponding content is represented (Kriete et al., 2013). The data of Frankland and Greene show that the stored content can be decoded from the brain area that represents its thematic role. If lmSTC represents this address, this implies that the address should include some aspects of the content represented there, similar to the proposed assembly projection model. Another possibility is that lmSTC corresponds to the addressed brain region. In this case, the data suggest that the address is relatively stable across the experiment. In conclusion, the data do not invalidate the indirection model. While its basic idea is quite distinct from the assembly projection concept, a hybrid model with aspects of both is another interesting possibility. We note that indirection models also often involve a disinhibitory gating mechanism (Kriete et al., 2013; for a more detailed discussion of this aspect, see O’Reilly, 2006). This provides further evidence that disinhibition is a powerful computational mechanism. The use of disinhibition is however quite different from our model. There, disinhibition is used to gate outputs of neural circuits, while in the assembly projection model, whole neural spaces are inhibited or disinhibited to suppress or enable activity and plasticity mechanisms there.

In anatomic binding models, different brain areas are regarded as distinct placeholders (similar to the neural spaces in this work). As each placeholder may have a unique representation for some content, anatomic binding models are consistent with the findings of Frankland and Greene (2015). A problem in anatomic binding models lies in providing a mechanism that allows to translate between the different representations. Building on previous work (Hayworth, 2012), a recent non-spiking model shows how external circuitry can be used to solve this problem (Hayworth and Marblestone, 2018). Each pair of placeholders requires multiple external processing circuits which are densely connected to the placeholders to allow transferring and comparing contents. The number of these processing circuits increases quadratically with the number of placeholders if contents should be transferable from and to every placeholder. While this may not be a problem if the number of placeholders is small, the model presented in this work avoids the need for additional circuitry altogether by using the content space as a central hub.

In neural blackboard architectures, one assumes that besides assemblies for content, there exist also assemblies for structural entities such as phrases, semantic roles, etc. Specialized circuits (so-called gating circuits and memory circuits) are then used to bind content to structure (van der Velde and de Kamps, 2006). This way, complex syntactic structures can be built. The concept shares some similarities with the assembly projection model. In particular, the “neural blackboard” is similar to our proposed content space. Assemblies there are then enriched with structural information. The way that this enrichment is implemented is quite orthogonal however. In neural blackboard architectures, activity is gated by specialized neural circuitry, while we propose that Hebbian plasticity leads to changes in connectivity and thus the emergence of structure encoding assemblies. It is not obvious how the findings in lmSTC can be interpreted from the perspective of a neural blackboard architecture, since structural assemblies are assumed to be fixed and independent of bound content. It is however possible that the bound content could influence the exact activity of the assembly, which could explain why content can be decoded from lmSTC (Frankland and Greene, 2015).

Vector symbolic architectures are a very powerful method to implement binding through neural networks (Plate, 1995; Eliasmith et al., 2012). Content as well as structural categories can be encoded as high-dimensional vectors, which could correspond to activity vectors of neurons in the brain. Rather complex mathematical operations can then be used to combine these vectors into composed representations where the structural information is bound together with the content. It has been shown that these operations can be implemented with spiking neural networks, and that higher-level cognitive functionality can result from such architectures (Eliasmith et al., 2012). While vector symbolic architectures necessitate quite precise synaptic connectivity patterns to implement the mathematical operations (Marcus et al., 2014b), we propose in this work that structure can be bound through plasticity processes in generic randomly connected networks.

In summary, the presented model combines the strengths of pointer-based (Zylberberg et al., 2013) and anatomic binding models (Hayworth and Marblestone, 2018). Like anatomic binding models (Hayworth and Marblestone, 2018), the dynamics of the proposed model match experimental data on the encoding of variables in human cortex (Frankland and Greene, 2015), but using the content space as a central hub eliminates the need to add circuitry as the number of variables increases. This is achieved by using a pointer-based mechanism (Kriete et al., 2013; Zylberberg et al., 2013).

### Experimental predictions

The validity of the assembly projection model could be tested experimentally, since it predicts quite unique network dynamics during mental operations. First, attachment of some structural category to a content employs disinhibition of a neural space. This could be implemented by the activation of inhibitory VIP cells which primarily target inhibitory neurons, or by neuromodulatory input. Similar disinhibition mechanisms would be observed during a recall of the content. Another prediction of the model is that the assembly projection that emerges in a neural space for some content should be similar to another one for the same content if it is re-established on a short or medium time scale. On the other hand, a significant modification of the assembly that encodes a concept will also modify the assembly projection that emerges in a neural space. Further, our model suggests that inactivation of an assembly projection to some content in neural space $S_{1}$ would not abolish the capability to create an attachment of the associated structural category $S_{1}$ to this content: if the trial that usually creates this linking is repeated, a new assembly projection in the neural space for $S_{1}$ can emerge. Finally, the model predicts that a mental task that requires to copy (or compare) the contents of a structural category $S_{1}$ to another structural category $S_{2}$ causes sequential activation (disinhibition) of the neural spaces $S_{1}$ and $S_{2}$ for categories.

The assembly projection model assumes that there is sufficient connectivity between the content space and neural spaces in both directions such that assemblies can be mutually excited. The most prominent language related areas in the human brain are Broca’s area and Wernicke’s area. In addition, it has been speculated that word-like elements are stored in the middle temporal cortex (Berwick and Chomsky, 2016), perhaps corresponding to the content space in our model. As discussed by Berwick and Chomsky (2016), these areas are connected by strong fiber pathways in adult humans, which could provide the necessary connectivity for the creation of assembly projections. The authors further point out that some of the pathways are missing in macaque monkeys and chimpanzees, possibly explaining the lack of human-like language capabilities in these species. We have proposed that lmSTC is one possible area hosting neural spaces for language-related structural categories. Synaptic pathways between STC and middle temporal cortex exist, consistent with the idea that the latter serves as a content space (data from Joshi et al., 2010, visualized in the human connectome project, Marcus et al., 2011; Laboratory of Neuro Imaging and Martinos Center for Biomedical Imaging, 2020).

### Model extensions and relation to other experimental work

We presented in this article a basic implementation of the assembly pointer concept. The model could be extended in several directions. The current model supports only recall of the most recently stored content of the neural space. However, by blocking activity of these neurons, previously stored content could be retrieved as well, since neural excitabilities and recurrent connections remain elevated for some extended time.

In a recent magnetoencephalography (MEG) study, Liu et al. (2019) found evidence for factorized structural representations of the position of a visual stimulus in a sequence. In the context of the assembly projection model, each of the four possible sequence positions in this experiment could be encoded by one neural space for position in a sequence, and appearance of an object at a position could result in an assembly projection to this neural space. Interestingly, they found that during spontaneous replay of these representations, the structural representation preceded the content representation by 40–60 ms. This timing is consistent with the timing of a recall in our model that starts from the neural space for sequence position. In this study, sequence position could be decoded first, at a point in time when stimulus identity (i.e., content) could not be decoded yet. We attribute this result to the quite coarse spatial resolution of MEG. Hence, position could be decodable from which neural space for sequence position is disinhibited and therefore shows activity, while the specific assembly pattern in this space (that is content-specific) might be indistinguishable.

In a recent modeling study, it was proposed that the hippocampus binds together spatial information represented in medial entorhinal cortex (EC) and content information from lateral EC (Whittington et al., 2018). Since this model is concerned with analog structural information, it is quite different from the assembly projection idea which works with structural categories. Nevertheless, it suggests an interesting extension of our model where the neural space is in addition driven by some other area that could, for example, represent an analog context variable such as time or space. In this case, the projection would not be a fully random assembly (defined by the network connectivity structure), but rather be formed by both connectivity structure and the momentary driving input. Hence, this assembly activation pattern would encode both the current content and the context variable.

In this article, we are presenting simulations in a realistic model that are compatible with experimental results of Frankland and Greene (2015) for binding words to roles in a sentence. Other recent experimental results (Zaccarella and Friederici, 2015; Ding et al., 2016; see also Friederici, 2017) seem to suggest that another assembly operation is at work in the processing of simple sentences: the operation MERGE proposed by Chomsky as the fundamental linguistic operator mediating the construction of sentences and phrases (Chomsky, 2014; Berwick and Chomsky, 2016). In related work (Papadimitriou and Vempala, 2018), it is shown that a MERGE operation on assemblies can indeed be realized by neurons in a simplified model. To illustrate MERGE within the assembly projection framework, suppose that there is a third neural space where verbs are projected, and an assembly for the word “hit” in content space has been projected to this space (Fig. 7). MERGE of the projected assemblies “hit” and “truck” in neural space would then create a new assembly in another subregion, which can be called phrase space (a brain area suspected to correspond to phrase space is the pars opercularis of Broca’s area, or BA 44; see Zaccarella and Friederici, 2015; Friederici, 2017; for a recent study suggesting such representations can be found in more anterior parts of prefrontal cortex, see also Frankland and Greene, 2019b). The resulting assembly would then represent the creation of the phrase “hit the truck,” and would have strong synaptic links to and from the two corresponding assemblies in neural space. Hence, MERGE entails the orchestration of two convergent streams, each consisting of two stacked projection operations, which together result in the flexible creation of a phrase assembly out of the two syntactic units (verb assembly and patient assembly). A second MERGE would then combine the agent and the phrase to create an assembly in sentence space (presumably the pars triangularis of Broca’s area, or BA 45) that represents the whole sentence “the ball hit the truck.” One may speculate that such stacking of assembly projections is what makes human language possible, and in fact it may be implicated in other cognitive functions such as deduction and planning. An interesting question in this regard is whether brains of non-human primates or even mice do implement mechanisms similar to assembly projection (thus enabling these animals to perform binding), and whether humans eventually evolved a more complex hierarchical variant of assembly projection. 

We have presented in this article assembly projections, a portable model for structured information representation in generic spiking neural networks. The comprehensive repertoire of operations on assemblies of neurons identified in the present paper, and the apparent effectiveness of these operations, seem to give credence to an emerging hypothesis that assemblies and their operations may underlie and enable many of the higher mental faculties of the human brain, such as language, planning, story-telling, and reasoning.

## Synthesis

Reviewing Editor: Tatyana Sharpee, The Salk Institute for Biological Studies Decisions are customarily a result of the Reviewing Editor and the peer reviewers coming together and discussing their recommendations until a consensus is reached. When revisions are invited, a fact-based synthesis statement explaining their decision and outlining what is needed to prepare a revision will be listed below. The following reviewer(s) agreed to reveal their identity: Adam Marblestone.

This work addresses a central question in cognitive science: what neural mechanisms enable the rapid combination of structure and content? It’s attempt to bridge high-level cognitive theory and neural implementation is admirable and ambitious. The idea of “assembly projections” for solving a variable-binding problem in neural circuits is novel and valuable, given that it can be implemented with simple Hebbian-like learning.

Reviewer 1:

The authors present a biologically plausible computational model that rapidly learns to encode structure-content combinations, such as the agent and patient of a sentence (who did what to whom?). The model is framed in terms of various recent findings in cognitive neuroscience; both in terms of the content representation (attributed to hippocampus), as well as the representation of variables, such as semantic role variables (left-mid superior temporal cortex). They show that this architecture is, in principle, capable of storing and retrieving bindings, “copying” representations between role-specific neural populations, and “comparing” the content of these populations.

Though I list a number of more specific points/questions below, my main concern revolves around the paper’s high-level framing and emphasis. Specifically, I see a tension between two ways of framing the authors’ modeling work (a) presenting the core idea of assembly projections and then referencing neural data that is generally consistent with this or (b) trying to actually explain existing neural data. The paper was somewhat inconsistent in its framing but I felt it tended to shift toward (b). However, I don’t know that this is the best approach, given that the empirical data in this area is limited, and moreover, the authors are inconsistent in what brain regions they are “explaining”. For example, the authors begin in the introduction by noting work on concept-codes in the hippocampus, but in the discussion, reference word-representations in middle temporal gyrus; a very different part of the temporal lobes. On the one hand, this would be fine if the paper were consistently and clearly about the general idea of assembly projections that could have multiple instantiations in different brain regions and different representational spaces. But that’s not really how it’s set up.

I think the paper would be considerably stronger if the primary emphasis/framing was on the formation of assembly projections for variable-binding as an abstract mechanism, with a somewhat more systematic comparison of this approach to other models. It can be highlighted (as the authors do) that this model is inspired by, and consistent with, empirical data regarding the hippocampus (or MTG if the authors prefer to focus on words) and lmSTC (Frankland & Greene, 2015).

Some of the particular places in which I think the modeling could be more complete are below.

More specific questions/comments

1. I had a hard time inferring which particular aspects of the model were critical for its performance and which were not. Given that we don’t know many of the actual empirical parameters in these regions (related to my general point above), I came away from the paper knowing that there exists some combination of parameters that lead to the formation of assembly projections that can perform these tasks. But what is essential? An existence proof like this has value in domains like this that aren’t well studied (like the current one), but I think the paper would be strengthened by more systematic presentation of the parameters and the results with different parameter combinations.

For example, here are some things I wondered about when reading the paper:

(a). The authors note that in the absence of input, the C and S spaces fire at 5.5 and 2.6. Hz respectively. What’s the motivation for this difference between the base firing rates between content and structural spaces? Is it meaningful? Do the results depend on this choice?

(b). Related in spirit, the authors write: “This initial learning phase consisted of 200 pattern presentations, where each presentation lasted for 200 ms followed by 200 ms of random activity of the input neurons (all firing at 12:5 Hz to have a roughly constant mean firing rate of all input neurons).”

Does it require 200 pattern presentations? This seems high compared to human learning rates. What happens with 1, 10, or 100 presentations?

(c.) The authors write: “This would mean that some neurons in the neural space are no longer available to form new projection assemblies, but this does not pose a problem if each neural space is sufficiently large.”

This seems like an important point; for a given number of items in the concept space, how large would the neural space have to be? Is the consistent with any empirical work? Does it make any predictions?

(d). Just out of curiosity, I wonder: does the model require that the neural spaces actually be anatomically constrained (i.e., local connections within neural spaces, but not between spaces?), or could role-specific spaces emerge given initially random connections?

(e). 100 Hz Poisson spike trains vs 0.1 Hz silent neurons. I’m not an expert here, but 0.1 hz seems surprisingly low (especially when contrasted with 100 hz stimulus evoked). If that is standard, perhaps a reference would be useful to at least signal that it’s appropriate. And related to my more general point, is it necessary that the firing rate be in these ranges?

2. Regarding the retrieval task, the authors write: “The increased excitability of recently activated neurons in S ensures that the most recently active assembly is activated which in turn activates the corresponding content through its (previously potentiated) feedback connections to content space C.”

Am I right to infer that this means that only the most recent binding can ever be retrieved? That is, if presented “John loves Mary” and then “Mary loves Tom", upon encountering “Mary loves Tom” the network would be unable retrieve the agent or patient of the preceding sentence (John loves Mary)? Is this a problem?

3. I thought the recall paradigm was in need of further justification. The authors write “A recall was regarded as a success if during the RECALL phase, at least 80% of the neurons which previously belonged to the concept assembly (measured after the assembly induction in the content space) were active (firing rate > 50 Hz), and if the number of erroneously active neurons did not exceed 20% of the original assembly size.”

It would be nice to see a decoding analysis here that showed that not only were a high proportion of the same cells active, but that this pattern uniquely identified the relevant concept, relative to the alternatives.

4. More generally, what is known about whether either of these regions connect to the region of lmSTC in which role spaces are purported to live? If the paper is going to be framed this way, there must be real constraints on the networks. At a minimum, there should be known bi-directional connections between the regions of interest....

5. I found the following statement confusing: “The data of Frankland and Greene, which shows that the stored content can be decoded from the brain area that represents its thematic role, speak against this model for language processing, as the address is in general unrelated to the stored content.”

I take the data of Frankland and Greene to show is that there is a reliable pattern of activity across instances for particular bindings. Thus, it seems the ability to decode the current “value” of the agent/patient variables could reflect either direct content or an indirect address-like representation (pointing to the content), so as long as that address is relatively stable across instances within the experiment. I don’t see how these empirical results adjudicate between these possibilities, but perhaps I’m missing it.

6. Relatedly, the Kriete et al. (2013) model (a pointer-based model), like the current model, also involves a disinhibitory gating mechanism (though of course, is based on very different neural circuits), and I thought warranted more careful attention, and perhaps even explicit comparison.

Reviewer 2:

MAJOR COMMENTS: I have a couple of major comments, which I don’t think necessitate further experiments (although that would certainly be nice), and in general I don’t want to delay publication of this important paper, but which may necessitate some additions or clarifications to the text and perhaps another explanatory / conceptual figure.

1) I am basically satisfied, at a high level, with the discussion and experiments here in the context of role-filler variable binding and simple sentence-type processing, and with the relationship of this to the motivating Frankland and Greene fMRI results. But in the introduction, abstract and conclusion, the authors apparently link their mechanism to something more general, namely to the idea of “structural knowledge", citing a paper by Behrens et al which includes, for instance, spatially or temporally organized / scaffolded / linked knowledge. Analog spatial or temporal coordinates or spaces, like those Behrens considers, though, are quite different from a small, discrete set of roles to which fillers can be attached, like the “subject” or “object” of a sentence. Also, we know more about how the former are coded, e.g., with place and grid fields. The authors should thus make an effort to more explicitly flesh out how assembly projections could apply to these other more spatial forms of “structural knowledge", or else restrict the scope to role-filler variable binding as such. To be clear, I think that linking assembly projections to structural knowledge more generally may well make sense and would be very exciting, but I’d like to see more detail on what the authors think that might look like if they are to extend beyond the discrete role-filler case. A few specifics:

a) While their major motivating experimental study for variable binding is, appropriately, the Frankland and Greene sentence fMRI paper, there are other studies that look at, for instance, temporal sequence order/position as a structural variable, and find really interesting physiologically-relevant things there. One is by Kurth Nelson et al,

https://www.sciencedirect.com/science/article/pii/S0092867419306403

in which they find: “A sensory code of object representations was preceded 50 ms by a code factorized into sequence position and sequence identity. We argue that this factorized representation facilitates the generalization of a previously learned structure to new objects.” This leads to several interesting aspects

a.i) Mechanistically, how would you propose to put temporal sequence order as a role variable in your framework? Should we have neural spaces for each potential position in the sequence? For each time? For just the current time?

a.ii) Given such a proposal, would we expect the decoding results to look like those of Kurth Nelson et al? An interesting aspect of which is: at certain timepoints, it seems they can decode position but *not* content (if I recall correctly). Is this then more consistent with a traditional pointer based binding? Or, would you predict that if they look with higher resolution, they would also be able to decode content whenever they can decode position? Or that the position information corresponds to which neural space is disinhibited while content corresponds to the pattern of activation within each such neural space? Perhaps content is decodable from the content space or areas feeding into it (distributed across the sensory areas of the brain), while position is decodable from which neural position space is allowed to be active. In any case, I can see interfacing with this paper as a fruitful way to extend the analysis to human neural data for cognitive tasks beyond the Frankland and Greene study, and thus an opportunity to make more predictions or reconcile any inconsistencies.

b) Representative of the more spatial school of structural knowledge representation in the brain is the recent paper by Whittington and Behrens called “Generalization of structural knowledge in the hippocampal-entorhinal system”. There, it is proposed that certain regions of the entorhinal cortex represent positional information which while others represent content information, and then these are merged together in hippocampus such that what we see as a place field is in fact the binding in hippocampus of content with structure from these two entorhinal input streams. Can this kind of idea be squared with the assembly projections idea?

c) In the present paper, the cell assemblies in the neural spaces for roles are essentially random. They are just whatever cells happen to be initially more strongly connected to a given content assembly or to fluctuate on when the assembly projection is first created. But you could imagine, instead, that the role assemblies are, for instance, place fields, or driven by place fields, or otherwise have some meaning or code for some structural variable. This could extend the model, perhaps, to analog variables like position, rather than discrete areas with one area for each role. Or perhaps this does not make sense. Please discuss.

2) I really like the discussion of the MERGE operation, but it needs a bit more self-contained explanation. Perhaps its own conceptual figure or sub-figure. What we are talking about is pointers to pointers, essentially, it seems. Given that this could be one of the key aspects of human uniqueness, according to this model, a bit more detail should be given as to what would really be required here. This would give opportunity to speculate a bit on how assembly projections fit into an overall picture of brain evolution. Do mice have assembly pointers? In which brain areas -- all over cortex, or just in specific temporal areas linked to hippocampus, or something else? How would MERGE and thus language have emerged through anatomical tweaking of a pre-existing structure that uses assembly pointers / projections for some *other* reason?

MINOR COMMENTS:

-They show that activity is decodable in the experiments analogous to the Frankland and Greene, but not necessarily at the fMRI multi-voxel pattern level of ∼1 second and 1 millimeter? Please comment.

-Explain the quadratic scaling in the context of the Hayworth + Marblestone paper. You need a thalamic relay / latch for each pair AB of areas you might want to copy information between. This could be made clearer. Also I am not sure if quadratic scaling is a problem if there are only a small number of specific role areas that need to be connected to content sources and to each other for copying or comparing. Do they think it needs to be a large number of role areas?

-"We study in this work not the standard form of a binding task, where several features are bound together to constitute an object representation (see, e.g. Plate, 1995; van der Velde and de Kamps, 2006; Eliasmith et al., 2012; Friederici and Singer, 2015, for models in that direction).” I find this a bit misleading as some of these papers do distinguish roles and fillers, I believe.

-At one point they refer to Marcus et al but then cite Zylberberg for the relevance of the COPY and COMPARE operations. It should be clarified a bit about the history of the COPY and COMPARE tasks.

-"operator mitigating the construction”: I think they mean “mediating"
